# Supplementary material for: Identification of a covert evolutionary pathway between two protein folds
Source: Nat Commun. 2023 Jun 1;14:3177. doi: 10.1038/s41467-023-38519-0 (PMC10235069; doi:10.1038/s41467-023-38519-0)
Supplement: Supplementary file 1 — Supplementary Information [file 41467_2023_38519_MOESM1_ESM.docx]

**Supplementary Information** for

**Identification of a covert evolutionary pathway between two protein folds**

Devlina Chakravarty^1^, Shwetha Sreenivasan^2^, Liskin Swint-Kruse^2^, and Lauren L. Porter^1,3,*^

^1^National Library of Medicine, National Center for Biotechnology Information, National Institutes of Health, Bethesda, MD 20894, USA

^2^Department of Biochemistry and Molecular Biology, The University of Kansas Medical Center, Kansas City, KS 66160

^3^National Heart, Lung, and Blood Institute, Biochemistry and Biophysics Center, National Institutes of Health, Bethesda, MD 20892, USA

^*^Corresponding Author: [lauren.porter@nih.gov](mailto:lauren.porter@nih.gov)

*Figure 1. Response regulator subfamilies cluster by C-terminal domain (CTD) architecture (left labels). Sequence comparisons between response regulators with experimentally determined structures were performed with jackhmmer^1^. PDB codes are indicated on the left and bottom axes of each panel. In these sequence identity matrices, structures with tetrahelical helix-turn-helix (HTH_4_) CTDS cluster in the upper left, whereas those with winged helix (wH) CTDs cluster in the lower right. The color bar indicates high (yellow) to low (black) sequence identity. (a) Sequence identity matrix of the NTDs lacks a strong distinction between response regulators with CTDs from the two fold families. (b). By contrast, the sequence identity matrix of the CTDs shows two distinct clusters that correspond to the different CTD architectures. Black dotted line separates HTH_4_ structures (above) and wH structures (below). Source data are provided as a Source Data file.*

*Figure 2. Box-and-whisker plots of log_10_(e-values) from jackhmmer searches of the 23 response regulators in the PDB. Sequence comparisons were made between (a) full-length sequences and (b) CTDs with (gray boxes) structures with the same fold and (white boxes) those with the other fold. The distributions of each white box in (a) were derived from N=12 (1A04, 1YIO, 3C3W, 4GVP, 4HYE, 4LDZ, 4YN8, 5F64, 5HEV, 5O8Y), 11 (1KGS, 1P2F, 2HQR, 4B09), 10 (5XSO, 2GWR, 4S04), 9 (2OQR), 8 (4KFC, 5VFA, 7LZ9), 7 (1YS6, 5ED4) e-values; the distributions of each black box came from N=11 (tetrahelical helix-turn-helix [HTH_4_], gray background) or N=12 (winged helix [wH], yellow background) e-values. The distributions of each white box in (b) were derived from N=24 (4GVP), 23 (3C3W, 5HEV), 22 (1A04, 4HYE), 20 (1YIO), 14 (4LDZ), 11 (1KGS), 10 (4B09), 7 (4YN8), 5 (1P2F, 2HQR, 2OQR), 4 (4S04), 3 (2GWR, 4KFC, 5VFA), 2 (1YS6, 5ED4), 1 (5O8Y, 7LZ9), 0 (5XSO) e-values; distributions from each black box in (b) were derived from N=26 (1KGS, 1P2F, 1YS6, 2GWR, 2HQR, 2OQR, 4B09, 4KFC, 4S04, 7LZ9), 25 (5VFA), 24 (5ED4), 19 (1A04, 1YIO, 3C3W, 4HYE, 4LDZ, 4YN8, 5F64, 5HEV, 5O8Y, 5XSO), 18 (4GVP) e-values. Each box bounds the interquartile range (IQR) of the data (first quartile, Q1 through third quartile, Q3); medians of each distribution are gray lines within each black box; lower whisker is the lowest datum above Q1-1.5*IQR; upper whisker is the highest datum below Q3 + 1.5*IQR. E-values < 5e-02 are considered significant. Note that the e-values of (b) are expected to be lower than those of (a) because the sequences are shorter. Source data are provided as a Source Data file.*

**

*Figure 3. MUSCLE^2^ alignment of 3,205 sequences also suggests a mutationally-driven secondary structure conversion. Secondary structure diagrams were generated from the structures of FixJ_PDB_ (black) and KdpE_PDB_ (yellow). The features of this diagram are analogous to those described in Fig 3a. Spaces between sequences show important changes: (1) orange linker insertion/deletion (2) fold conversion and possible β-sheet insertion/deletion. Source data are provided as a Source Data file.*

*
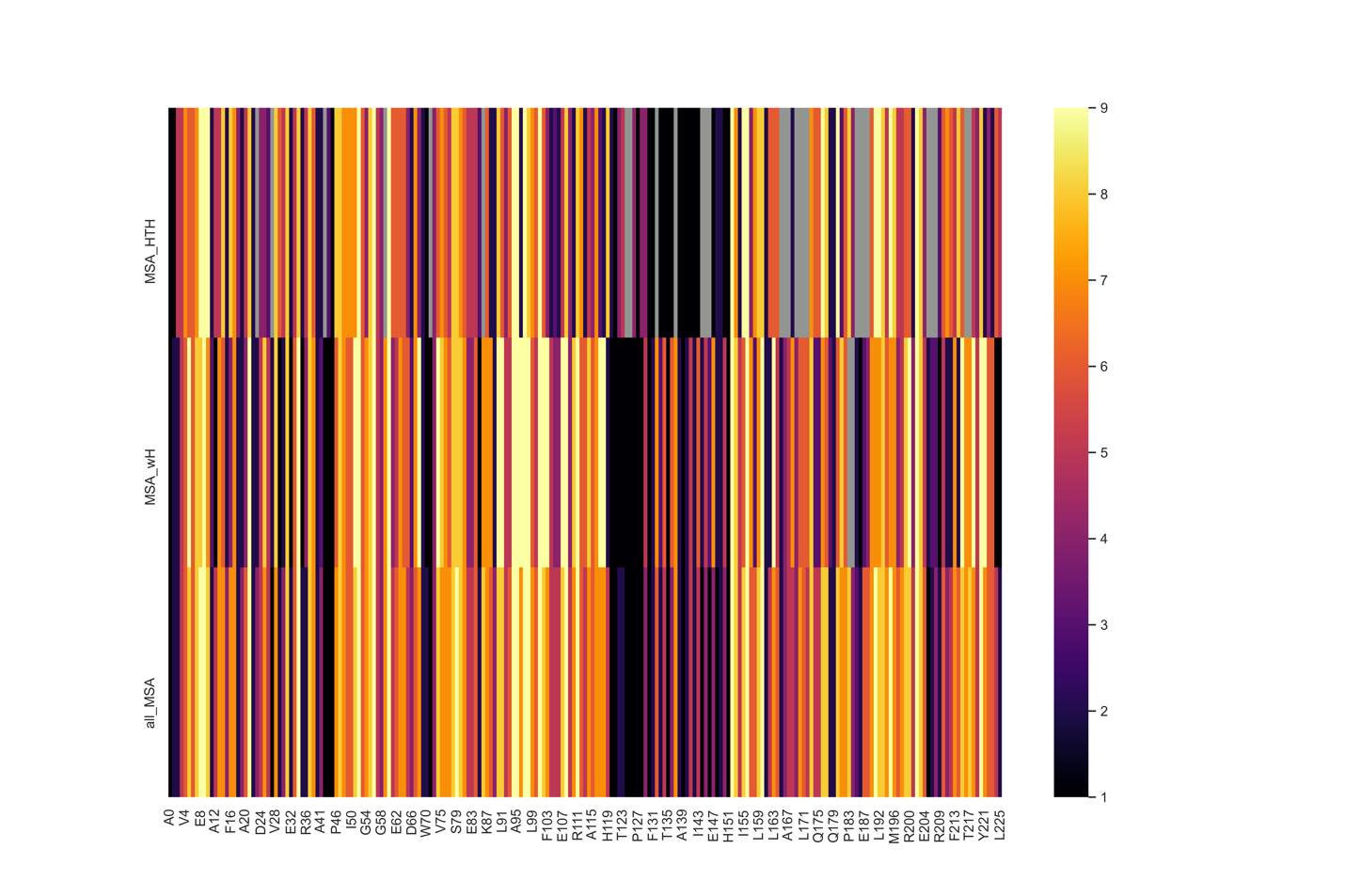
*

*Figure 4. Evolutionary rates of only tetrahelical helix-turn-helix (HTH_4_) (664, top), only winged (wH) (2541, middle), and all 3205 sequences of the cross-family multiple sequence alignment (MSA, bottom) of Figure 3b. These rates were calculated using ConSurf^3^ to analyze the Clustal Omega^4^ alignment and the consensus tree. Position numbers at the bottom of the plot correspond to those of KdpE_PDB_ (PBD ID: 4KFC, chain A). The conservation scale (color bar) ranges from 1 (not conserved, black) to 9 (highly conserved, yellow); gray represents alignment gaps. The greater number of wH sequences (Figure 3) probably biases the results of the “all_MSA” calculation. Conservation patterns in the N-terminal domains (positions 0-119) of all 3 sets of sequences show similar rates in similar positions. In both subfamilies, the inter-domain linker shows more rapid evolution (darker colors) than either the NTD or CTD. By contrast, conservation patterns in the C-terminal domains differ. Most notably, several positions in the wing of the wH sequences (positions 213-225) are highly conserved (score of 9), whereas corresponding positions in the HTH_4_ evolve more rapidly (scores of 1-6). Source data are provided as a Source Data file.*

**
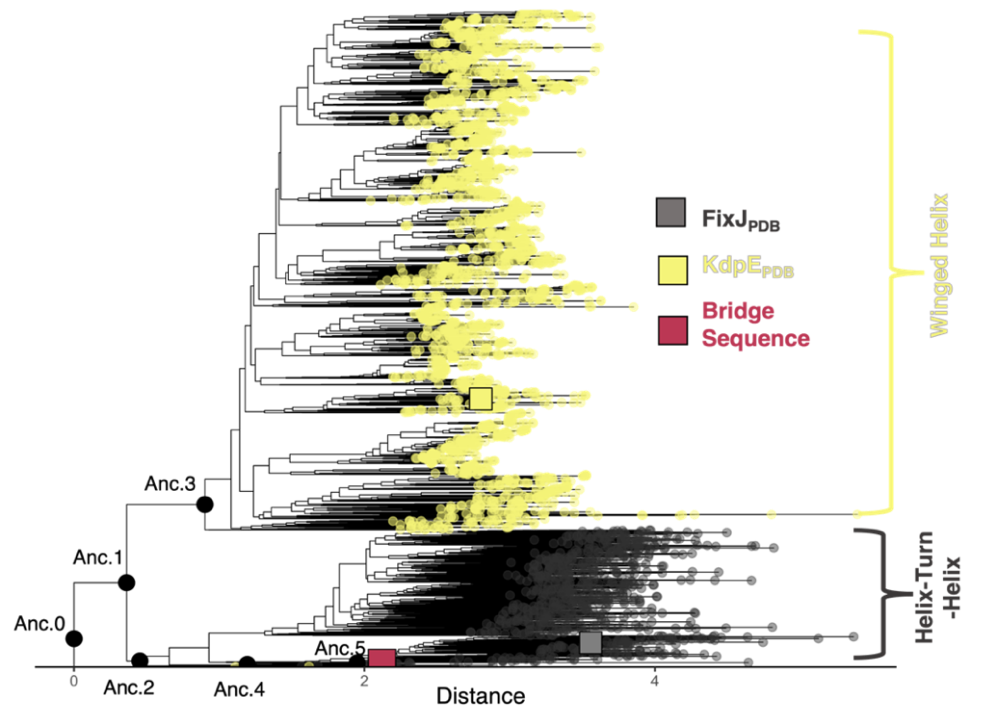
***
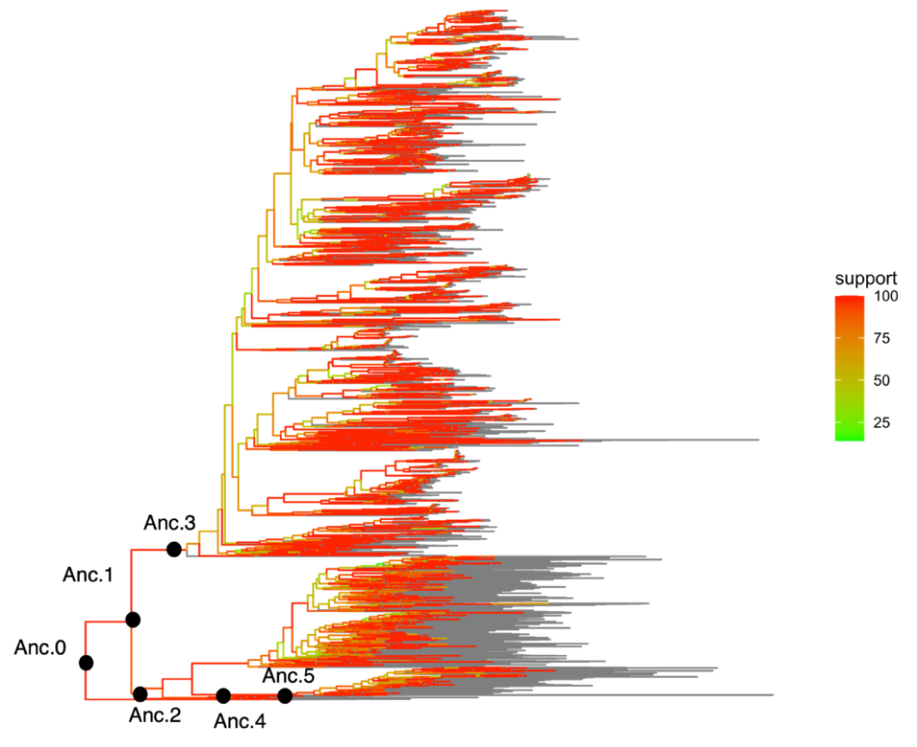
*

**b**

**a**

*Figures 5. Further phylogenetic analyses of response regulator proteins. (a) Unrooted consensus tree built after ultrafast bootstrapping using the maximum likelihood (ML) tree (Figure 3) as input. The Robinson-Foulds distance (which denotes the number of splits needed to convert one tree to another) between this tree and the ML tree in Figure 3 is 127, indicating 98% similarity between the two trees, and confirming the accuracy of the tree shown in Figure 3b. The bridge sequences in this unrooted consensus tree can also lie between the fold families if all branches connected at Anc. 2 are flipped by 180°. Thus, we show the ML tree in Figure 3b to highlight that the branch containing the bridge sequences can adjoin both fold families. Nodes annotated as tetrahelical helix-turn-helix (HTH_4_) and winged-helix (wH) are colored yellow and gray, respectively. This consensus tree was used for ancestral reconstruction (Figure 4); ancestral nodes are marked as black circles. (b) Bootstrapping support shown on the consensus tree after performing ultrafast bootstrapping for 1000 replicates at 500 iterations. Nodes are color-coded by the level of support shown in the color bar. Most branches are well supported with values > 80%. Importantly, all ancestral nodes (black circles) are supported with values > 95%. Locations of the unsupported gray branches are ambiguous.*

*
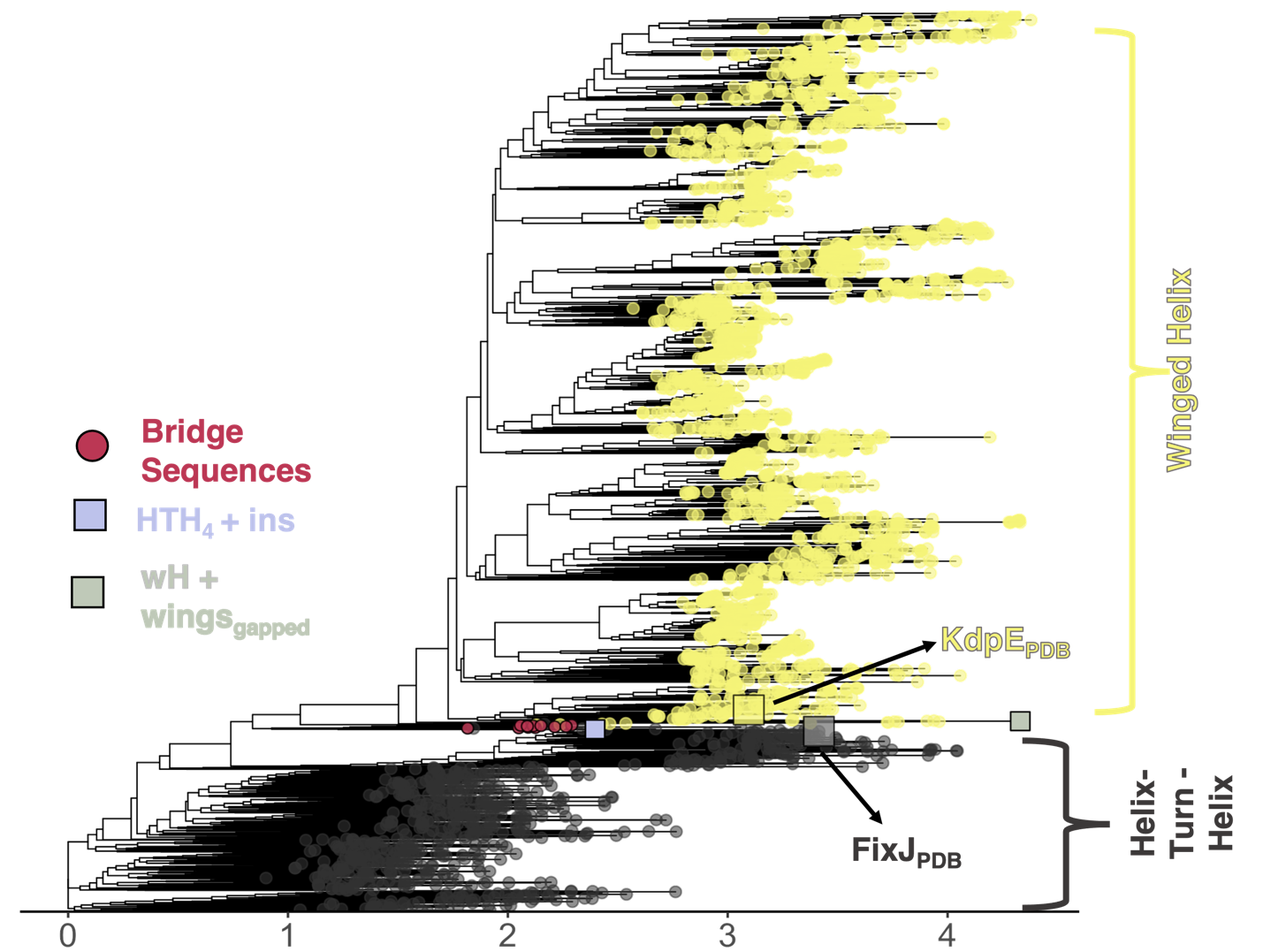
*

**Distance**

*Figure 6. Maximum-likelihood phylogenetic trees constructed from MUSCLE alignment also suggest an evolutionary path between response regulators with tetrahelical helix-turn-helix (HTH_4_) and winged helix (wH) folds. Sequences with C-terminal domains (CTDs) annotated as HTH_4_/wH from NCBI protein records are gray/yellow. The clade containing the 12 identified bridging sequences is highlighted in pink. HTH_4­­­_insert_ provides an example of an annotated HTH_4_ sequence whose linker length was similar to wH; wH_wings_gapped_ provides an example of a wH sequence with a 2-residue deletion similar to those found in >99% of the C-terminal helices of aligned HTH_4_ sequences. Distance units are arbitrary, though sequences further in space have more distant evolutionary relationships.*

*Figures 7. Selection of likely tree rootings was based on the p-value of approximately unbiased (pAU) test (one-sided). Frequencies (a) and ranks (b) of probable tree rootings from the approximately unbiased test (pAU) of the tree shown in Figure 3b and Supplementary Figure 5. Although most rootings were highly improbable, 18of 6393 had a pAU ≥0.8 (b, red box), suggesting likely rootings. Source data are provided as a Source Data file.*

*
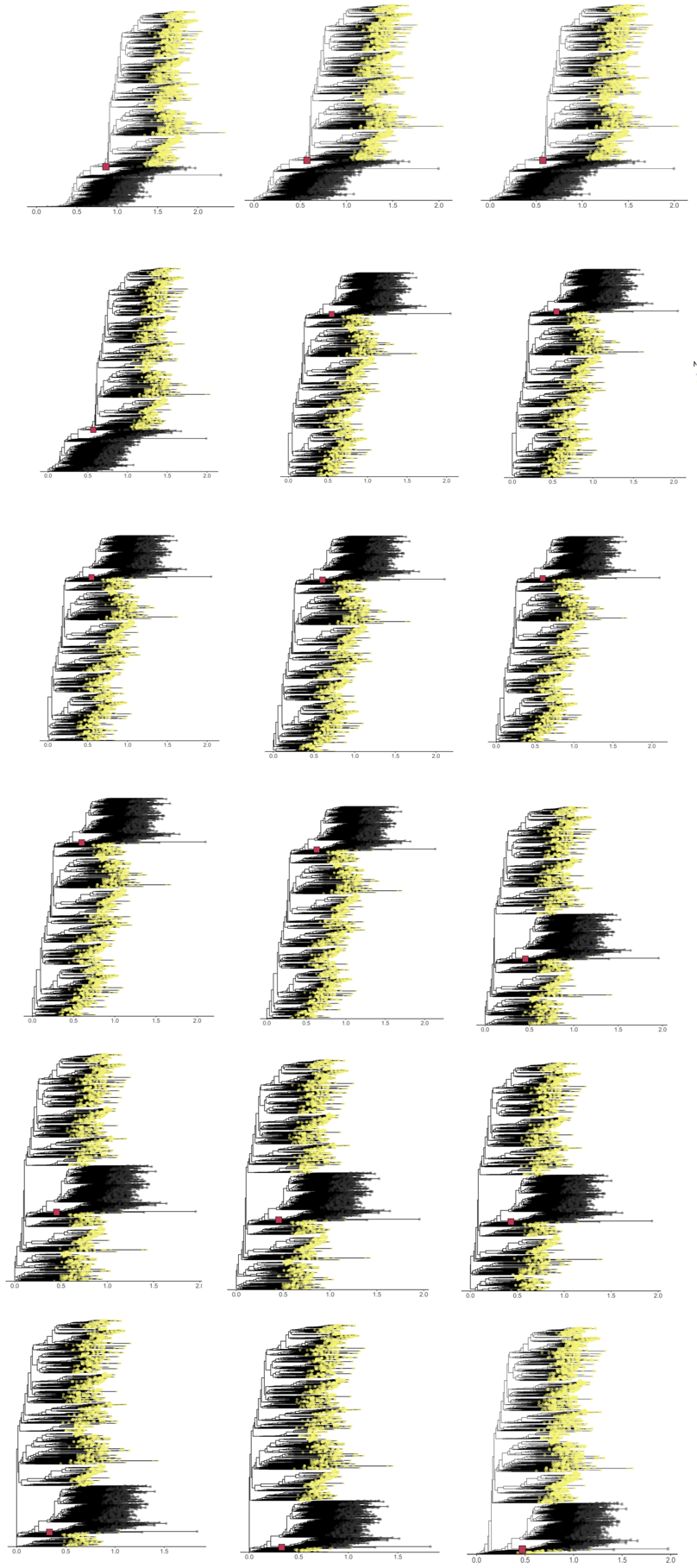
*

*Figure 8. Ancestors to the branch containing the 12 bridge sequences may have served as the evolutionary intermediates between helix-turn-helix and winged helix folds. The branches with bridge sequences (pink square) adjoin branches with helix-turn-helix (gray) and winged helix (yellow) sequences in all 18 phylogenetic trees with most probable rootings.*

*
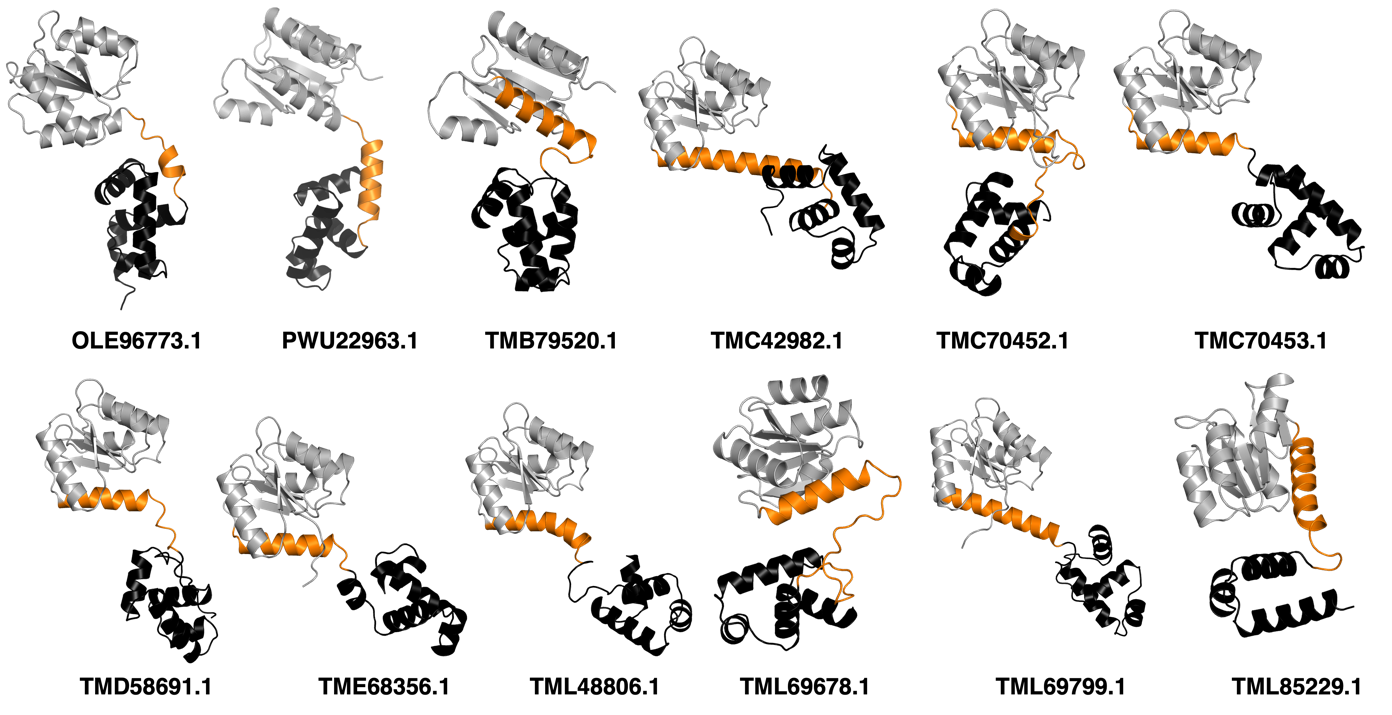
*

*Figure 9. AlphaFold2 structure predictions for the 12 bridge sequences. In all 12 cases, the C-terminal DNA-binding domains (black) are predicted to assume helix-turn-helix folds, suggesting that evolved fold switching may have occurred in ancestral sequences. The N-terminal receiver domains are gray, and the linkers are orange. NCBI Protein database accession codes are shown below each model; their sequences are reported in* ***Supplementary*** ***Table 4****.*

*Table 1. PSI-BLAST^5^ searches of FixJ_PDB_ sequence (query, PDB ID: 5XSO, chain A) against all the sequences found in the PDB identified dozens of evolutionarily-related sequences, the top 20 of which are shown below. The three columns show the PDB ID, e-value, and alignment, respectively. The C-terminal domain (CTD) of FixJ_PDB_ assumes a helix-turn-helix fold. White rows indicate alignments with other response regulators with CTD helix-turn-helix folds. Gray rows indicate alignments with response regulators with CTD winged helix folds.*

| PDB ID | e-value | Alignment |
| --- | --- | --- |
| 5XSO | 7e-55 | Query 1 MTTKGHIYVIDDDAAMRDSLNFLLDSAGFGVTLFDDAQAFLDALPGLSFGCVVSDVRMPG  60           MTTKGHIYVIDDDAAMRDSLNFLLDSAGFGVTLFDDAQAFLDALPGLSFGCVVSDVRMPG  Sbjct 11 MTTKGHIYVIDDDAAMRDSLNFLLDSAGFGVTLFDDAQAFLDALPGLSFGCVVSDVRMPG  70    Query 61 LDGIELLKRMKAQQSPFPILIMTGHGDVPLAVEAMKLGAVDFLEKPFEDDRLTAMIESAI  120           LDGIELLKRMKAQQSPFPILIMTGHGDVPLAVEAMKLGAVDFLEKPFEDDRLTAMIESAI  Sbjct 71 LDGIELLKRMKAQQSPFPILIMTGHGDVPLAVEAMKLGAVDFLEKPFEDDRLTAMIESAI  130    Query 121 RQAEPAAKSEAVAQDIAARVASLSPRERQVMEGLIAGLSNKLIAREYDISPRTIEVYRAN  180           RQAEPAAKSEAVAQDIAARVASLSPRERQVMEGLIAGLSNKLIAREYDISPRTIEVYRAN  Sbjct 131 RQAEPAAKSEAVAQDIAARVASLSPRERQVMEGLIAGLSNKLIAREYDISPRTIEVYRAN  190    Query 181 VMTKMQANSLSELVRLAMRAGMLND  205           VMTKMQANSLSELVRLAMRAGMLND  Sbjct 191 VMTKMQANSLSELVRLAMRAGMLND  215 |
| 1YIO | 2e-48 | Query 1 MTTKGHIYVIDDDAAMRDSLNFLLDSAGFGVTLFDDAQAFLDALPGLSFGCVVSDVRMPG  60  MT K ++V+DDD ++R+ L  LL SAGF V  FD A  FL+   GC+V D+RMPG  Sbjct 1 MTAKPTVFVVDDDMSVREGLRNLLRSAGFEVETFDCASTFLEHRRPEQHGCLVLDMRMPG  60    Query 61 LDGIELLKRMKAQQSPFPILIMTGHGDVPLAVEAMKLGAVDFLEKPFEDDRLTAMIESAI  120           + GIEL +++ A PI+ +T HGD+P+ V AMK GA++FL KPFE+  L   IE  +  Sbjct 61 MSGIELQEQLTAISDGIPIVFITAHGDIPMTVRAMKAGAIEFLPKPFEEQALLDAIEQGL  120    Query 121 RQAEPAAKSEAVAQDIAARVASLSPRERQVMEGLIAGLSNKLIAREYDISPRTIEVYRAN  180          +  ++  + +SL+ RE+QV++  I GL NK IA E  I+  T++V+R N  Sbjct 121 QLNAERRQARETQDQLEQLFSSLTGREQQVLQLTIRGLMNKQIAGELGIAEVTVKVHRHN  180    Query 181 VMTKMQANSLSELVRLAMR  199           +M K+   SL+ LV L  +  Sbjct 181 IMQKLNVRSLANLVHLVEK  199 |
| 4HYE | 1e-42 | Query  5 GHIYVIDDDAAMRDSLNFLL--DSAGFGVTLFDDAQAFLDALPGLSFGCVVSDVRMPGLD  62             + V +D + +RD++  LL    V + Q  +  L   S + DV MP  Sbjct  22   MKVLVAEDQSMLRDAMCQLLTLQPDVESVLQAKNGQEAIQLLEKESVDIAILDVEMPVKT  81    Query  63   GIELLKRMKAQQSPFPILIMTGHGDVPLAVEAMKLGAVDFLEKPFEDDRLTAMIESAIRQ  122           G+E+L+ +++++ ++++T      A+K G   ++ K  L   + + +  Sbjct  82   GLEVLEWIRSEKLETKVVVVTTFKRAGYFERAVKAGVDAYVLKERSIADLMQTLHTVLEG  141    Query  123  AEPAAKSEAVAQDIAARVASLSPRERQVMEGLIAGLSNKLIAREYDISPRTIEVYRANVM  182            +  + + + +  R   L+ +E  V++G+  GLSN+ IA +  +S  TI  Y  N++  Sbjct  142  RKEYSPE--LMEMVMTRPNPLTEQEIAVLKGIARGLSNQEIADQLYLSNGTIRNYVTNIL  199    Query  183  TKMQANSLSELVRLAMRAGML  203           +K+ A + +E   +A  +G L  Sbjct  200  SKLDAGNRTEAANIAKESGWL  220 |
| 4ZMR | 2e-42 | Query  5 GHIYVIDDDAAMRDSLNFLL--DSAGFGVTLFDDAQAFLDALPGLSFGCVVSDVRMPGLD  62             + V +D + +RD++  LL    V + Q  +  L   S + DV MP  Sbjct  9 MKVLVAEDQSMLRDAMCQLLTLQPDVESVLQAKNGQEAIQLLEKESVDIAILDVEMPVKT  68    Query  63  GIELLKRMKAQQSPFPILIMTGHGDVPLAVEAMKLGAVDFLEKPFEDDRLTAMIESAIRQ  122           G+E+L+ +++++ ++++T      A+K G   ++ K  L   + + +  Sbjct  69  GLEVLEWIRSEKLETKVVVVTTFKRAGYFERAVKAGVDAYVLKERSIADLMQTLHTVLEG  128    Query  123  AEPAAKSEAVAQDIAARVASLSPRERQVMEGLIAGLSNKLIAREYDISPRTIEVYRANVM  182            +  + + + + R   L+ +E  V++G+  GLSN+ IA +  +S  TI  Y  N++  Sbjct  129  RKEYSPE--LMEMVMTRPNPLTEQEIAVLKGIARGLSNQEIADQLYLSNGTIRNYVTNIL  186    Query  183  TKMQANSLSELVRLAMRAGML  203           +K+ A + +E   +A  +G L  Sbjct  187  SKLDAGNRTEAANIAKESGWL  207 |
| 5ED4 | 4e-41 | Query  2 TTKGHIYVIDDDAAMRDSLNFLLDSAGFGVTLFDDAQAFLDALPGLSFGCVVSDVRMPGL  61  T +  + V+DD+A + + L+  L   GF V + LD     V+ DV MPG+  Sbjct 21 TPEARVLVVDDEANIVELLSVSLKFQGFEVYTATNGAQALDRARETRPDAVILDVMMPGM  80    Query  62 DGIELLKRMKAQQSPFPILIMTGHGDVPLAVEAMKLGAVDFLEKPFEDDRLTAMIESAIR  121           DG  +L+R++A P L +T +   +  + LG  D++ KPF  + + A +   +R  Sbjct  81 DGFGVLRRLRADGIDAPALFLTARDSLQDKIAGLTLGGDDYVTKPFSLEEVVARLRVILR  140    Query  122 QA----EPAAKSEAVAQDIAARVA-----------SLSPRERQVMEGLIAGLSNK-----  161          +A +  DI            SLSP E  ++   +  Sbjct  141  RAGKGNKEPRNVRLTFADIELDEETHEVWKAGQPVSLSPTEFTLLRYFVINAGTVLSKPK  200    Query  162  ----LIAREYDISPRTIEVYRANVMTKMQANSLSELVRLAMRAGML  203               +   ++  +E Y + +  K+   L+  G +  Sbjct  201  ILDHVWRYDFGGDVNVVESYVSYLRRKIDTGEK-RLLHTLRGVGYV  245 |
| 5HEV | 9e-41 | Query  4 KGHIYVIDDDAAMRDSLNFLLD-SAGFGVT-LFDDAQAFLDALPGLSFGCVVSDVRMPGL  61              + ++DD  +R  ++  L   V ++ +   + L ++ D+ M  +  Sbjct  1 MIKVLLVDDHEMVRLGVSSYLSIQEDIEVIGEAENGRQGYEKAMALRPDVILMDLVMEEM  60    Query  62   DGIELLKRMKAQQSPFPILIMTGHGDVPLAVEAMKLGAVDFLEKPFEDDRLTAMIESAIR  121           DGIE  K +    I+I+T   D  A++ GA  +L K  +   I +  R  Sbjct  61   DGIESTKAILKDWPKAKIIIVTSFIDDEKVYPAIEAGAAGYLLKTSTAHEIADAIRATQR  120    Query  122  --QAEPAAKSEAVAQDIAARVAS-----LSPRERQVMEGLIAGLSNKLIAREYDISPRTI  174             +  +  + + ++ R    L+ RE +++  +  G SN+ IA E  I+ +T+  Sbjct  121  GERVLEPEVTTKMMEKMSRRNDPVLHEELTNRENEILMLISEGKSNQEIADELFITLKTV  180    Query  175  EVYRANVMTKMQANSLSELVRLAMRAGML  203           + + +N++ K++ ++ A + G++  Sbjct  181  KTHVSNILAKLEVEDRTQAAIYAFKHGLV  209 |
| 1KGS | 3e-40 | Query  4 KGHIYVIDDDAAMRDSLNFLLDSAGFGVTLFDDAQAFLDALPGLSFGCVVSDVRMPGLDG  63              + V++D+  + D +   L F V +  D +      F  V+ D+ +P  DG  Sbjct  2 NVRVLVVEDERDLADLITEALKKEXFTVDVCYDGEEGXYXALNEPFDVVILDIXLPVHDG  61    Query  64 IELLKRMKAQQSPFPILIMTGHGDVPLAVEAMKLGAVDFLEKPFEDDRLTAMIESAIRQA  123            E+LK  +  P+L +T   DV   V+ +  GA D+L KPF+   L A + + IR+  Sbjct  62 WEILKSXRESGVNTPVLXLTALSDVEYRVKGLNXGADDYLPKPFDLRELIARVRALIRRK  121    Query  124 EPAAKSEAVAQDI-----------AARVASLSPRERQVMEGLIAGLSNKLIAREY-----  167             +  ++ V  D+        ++   L+ +E Q++E L+   +  +   E  Sbjct  122 SESKSTKLVCGDLILDTATKKAYRGSKEIDLTKKEYQILEYLVXNKNRVVTKEELQEHLW  181    Query  168  ----DISPRTIEVYRANVMTKMQANSLSELVRLAMRAGML  203               ++ +  +  N+  K+  +++  G +  Sbjct  182  SFDDEVFSDVLRSHIKNLRKKVDKGFKKKIIHTVRGIGYV  221 |
| 4GVP | 4e-40 | Query  4 KGHIYVIDDDAAMRDSLNFLLD-SAGFGVT-LFDDAQAFLDALPGLSFGCVVSDVRMPGL  61              +  +DD   +R  ++  L   +   V   +  + L ++ D+ M  +  Sbjct  1 TIKVLFVDDHEMVRIGISSYLSTQSDIEVVGEGASGKEAIAKAHELKPDLILMDLLMEDM  60    Query  62   DGIELLKRMKAQQSPFPILIMTGHGDVPLAVEAMKLGAVDFLEKPFEDDRLTAMIESAIR  121           DG+E   ++K   +L++T   +  A+ G   ++ K  +   + R  Sbjct  61   DGVEATTQIKKDLPQIKVLMLTSFIEDKEVYRALDAGVDSYILKTTSAKDIADAVRKTSR  120    Query  122  QAEPAAKSEAV-----AQDIAARVASLSPRERQVMEGLIAGLSNKLIAREYDISPRTIEV  176                     V  +  A L+ RE +++  +  G SN+ IA I+ +T++  Sbjct  121  GESVFEPEVLVKMRNRMKKRAELYEMLTEREMEILLLIAKGYSNQEIASASHITIKTVKT  180    Query  177  YRANVMTKMQANSLSELVRLAMRAGML  203           + +N+++K++ ++ V A + ++  Sbjct  181 HVSNILSKLEVQDRTQAVIYAFQHNLI  207 |
| 4LDZ | 1e-39 | Query  3 TKGHIYVIDDDAAMRDSLNFLLD-SAGFGVTL-FDDAQAFLDALPGLSFGCVVSDVRMPG  60           +   I++ +D   +  +L  LL+  V   Q  +D +    + D+ MPG  Sbjct  4 SMISIFIAEDQQMLLGALGSLLNLEDDMEVVGKGTTGQDAVDFVKKRQPDVCIMDIEMPG  63    Query  61 LDGIELLKRMKAQQSPFPILIMTGHGDVPLAVEAMKLGAVDFLEKPFEDDRLTAMIESAI  120             G+E  + +K   +   I+I+T      A+K G   +L K +L   I S +  Sbjct  64 KTGLEAAEELK--DTGCKIIILTTFARPGYFQRAIKAGVKGYLLKDSPSEELANAIRSVM  121    Query  121 RQAEPAAKSEAVAQDIAARVASLSPRERQVMEGLIAGLSNKLIAREYDISPRTIEVYRAN  180               A + +D+ + L+ RE++V+E +  G + K IA+E  I   T+  Y +  Sbjct  122  NGKRIYAPE--LMEDLYSEANPLTDREKEVLELVADGKNTKEIAQELSIKSGTVRNYISM  179    Query  181  VMTKMQANSLSELVRLAMRAGM  202           ++ K++  +  E +  +   G  Sbjct  180  ILEKLEVKNRIEAITRSKEKGW  201 |
| 4KFC | 6e-39 | Query  3 TKGHIYVIDDDAAMRDSLNFLLDSAGFGVTLFDDAQAFLDALPGLSFGCVVSDVRMPGLD  62              ++ +++D+ A+R  L   L+  G  V   +  Q  L      ++ D+ +P  D  Sbjct  2 AMANVLIVEDEQAIRRFLRTALEGDGMRVFEAETLQRGLLEAATRKPDLIILDLGLPDGD  61    Query  63   GIELLKRMKAQQSPFPILIMTGHGDVPLAVEAMKLGAVDFLEKPFEDDRLTAMIESAIRQ  122           GIE ++ ++   S  P+++++   + + A+  GA D+L KPF L A +  A+R+  Sbjct  62   GIEFIRDLRQ-WSAVPVIVLSARSEESDKIAALDAGADDYLSKPFGIGELQARLRVALRR  120    Query  123  A------EPAAKSEAVAQDIAAR-------VASLSPRERQVMEGLIAGLSNKLIAREYD-  168                  +P  K   V D+AAR      L+P E +++  L+  L  R+  Sbjct  121  HSATTAPDPLVKFSDVTVDLAARVIHRGEEEVHLTPIEFRLLAVLLNNAGKVLTQRQLLN  180    Query  169  --------ISPRTIEVYRANVMTKMQANS-LSELVRLAMRAGM  202                        + +Y  ++  K++ +     A G  Sbjct  181  QVWGPNAVEHSHYLRIYMGHLRQKLEQDPARPRHFITATGIGY  223 |
| 3R0J | 1e-38 | Query  2 TTKGHIYVIDDDAAMRDSLNFLLDSAGFGVTLFDDAQAFLDALPGLSFGCVVSDVRMPGL  61           T +  + V+DD+A + + L+  L   GF V + LD     V+ DV  PG  Sbjct  21   TPEARVLVVDDEANIVELLSVSLKFQGFEVYTATNGAQALDRARETRPDAVILDVXXPGX  80    Query  62   DGIELLKRMKAQQSPFPILIMTGHGDVPLAVEAMKLGAVDFLEKPFEDDRLTAMIESAIR  121           DG  +L+R++A P L +T +   +  + LG  D++ KPF  + + A +   +R  Sbjct  81   DGFGVLRRLRADGIDAPALFLTARDSLQDKIAGLTLGGDDYVTKPFSLEEVVARLRVILR  140    Query  122  QA----EPAAKSEAVAQDIAARVA-----------SLSPRERQVMEGLIAGLSNK-----  161           +A +      DI            SLSP E  ++   +  Sbjct  141  RAGKGNKEPRNVRLTFADIELDEETHEVWKAGQPVSLSPTEFTLLRYFVINAGTVLSKPK  200    Query  162  ----LIAREYDISPRTIEVYRANVMTKMQANSLSELVRLAMRAGML  203              +   ++  +E Y + +  K+   L+  G +  Sbjct  201  ILDHVWRYDFGGDVNVVESYVSYLRRKIDTGEK-RLLHTLRGVGYV  245 |
| 3Q9S | 2e-38 | Query  6 HIYVIDDDAAMRDSLNFLLDSAGFGVTLFDDAQAFLDALPGLSFGCVVSDVRMPGLDGIE  65            I VI+DD  + + L   L  AG+ V   D A   L      ++ D+ +P  DG +  Sbjct  39   RILVIEDDHDIANVLRXDLTDAGYVVDHADSAXNGLIKAREDHPDLILLDLGLPDFDGGD  98    Query  66   LLKRMKAQQSPFPILIMTGHGDVPLAVEAMKLGAVDFLEKPFEDDRLTAMIESAIRQAEP  125           +++R++   S  PI+++T V   V  + LGA D+L KPF  D L A ++  +RQ  Sbjct  99   VVQRLRKN-SALPIIVLTARDTVEEKVRLLGLGADDYLIKPFHPDELLARVKVQLRQRTS  157    Query  126  AAKSEAVAQDIAAR--------VASLSPRERQVMEGLIA---------GLSNKLIAREYD  168            + S     +       LSP+E  ++  LI       +  ++  Sbjct  158  ESLSXGDLTLDPQKRLVTYKGEELRLSPKEFDILALLIRQPGRVYSRQEIGQEIWQGRLP  217    Query  169  ISPRTIEVYRANVMTKMQANSLSELVRLAMR  199                ++V+ AN+  K++  L+R  Sbjct  218  EGSNVVDVHXANLRAKLRDLDGYGLLRTVRG  248 |
| 4KNY | 2e-38 | Query  3 TKGHIYVIDDDAAMRDSLNFLLDSAGFGVTLFDDAQAFLDALPGLSFGCVVSDVRMPGLD  62              ++ +++ D+ A+R  L   L+  G  V   +  Q  L      ++ D+ +P  D  Sbjct  2 AMANVLIVEDEQAIRRFLRTALEGDGMRVFEAETLQRGLLEAATRKPDLIILDLGLPDGD  61    Query  63   GIELLKRMKAQQSPFPILIMTGHGDVPLAVEAMKLGAVDFLEKPFEDDRLTAMIESAIRQ  122           GIE ++ ++   S  P+++++   + + A+  GA D+L KPF L A +  A+R+  Sbjct  62   GIEFIRDLRQ-WSAVPVIVLSARSEESDKIAALDAGADDYLSKPFGIGELQARLRVALRR  120    Query  123  A------EPAAKSEAVAQDIAAR-------VASLSPRERQVMEGLIAGLSNKLIAREYD-  168                  +P  K   V  D+AAR      L+P E +++  L+  L  R+  Sbjct  121  HSATTAPDPLVKFSDVTVDLAARVIHRGEEEVHLTPIEFRLLAVLLNNAGKVLTQRQLLN  180    Query  169  --------ISPRTIEVYRANVMTKMQANSLSELVRLAMRAGM  202                        + +Y  ++  K++ +     G+  Sbjct  181  QVWGPNAVEHSHYLRIYMGHLRQKLE-QDPARPRHFITETGI  221 |
| 4S04 | 4e-38 | Query  5 GHIYVIDDDAAMRDSLNFLLDSAGFGVTLFDDAQAFLDALPGLSFGCVVSDVRMPGLDGI  64             I VI+DDA +   L   + S G+   A +L +  +V D+ +P  DG+  Sbjct  1 MKILVIEDDALLLQGLILAMQSEGYVCDGVSTAHEAALSLASNHYSLIVLDLGLPDEDGL  60    Query  65  ELLKRMKAQQSPFPILIMTGHGDVPLAVEAMKLGAVDFLEKPFEDDRLTAMIESAIRQAE  124             L RM+ ++   P+LI+T +   +  +  GA D+L KPF  + L A I + +R+  Sbjct  61  HFLSRMRREKMTQPVLILTARDTLEDRISGLDTGADDYLVKPFALEELNARIRALLRRHN  120    Query  125 PAAKSEAVAQDIAARVA-----------SLSPRERQVMEGLIAGLS---------NKLIA  164               +E ++ V         L+P+E  ++  L+         N + +  Sbjct  121 NQGDNEISVGNLRLNVTRRLVWLGETALDLTPKEYALLSRLMMKAGSPVHREILYNDIYS  180    Query  165 REYDISPRTIEVYRANVMTKMQANSLSELVR  195            + + +  T+EV+  N+  K+   S VR  Sbjct  181 GDNEPATNTLEVHIHNLREKIG-KSRIRTVR  210 |
| 2OQR | 4e-38 | Query  2 TTKGHIYVIDDDAAMRDSLNFLLDSAGFGVTLFDDAQAFLDALPGLSFGCVVSDVRMPGL  61               + +++D+ ++ D L FLL   GF  T+  D  A L      V+ D+ +PG+  Sbjct  2 AMATSVLIVEDEESLADPLAFLLRKEGFEATVVTDGPAALAEFDRAGADIVLLDLMLPGM  61    Query  62 DGIELLKRMKAQQSPFPILIMTGHGDVPLAVEAMKLGAVDFLEKPFEDDRLTAMIESAIR  121            G ++ K+++A+ S  P++++T    V  ++LGA D++ KP+ L A I + +R  Sbjct  62 SGTDVCKQLRAR-SSVPVIMVTARDSEIDKVVGLELGADDYVTKPYSARELIARIRAVLR  120    Query  122 QA--EPAAKSEAVAQDIAARVA-------------SLSPRERQVMEGLIAGLSNKLIARE  166           +   + +  S+ V + R+          +L  +E  ++E L+  L  +  Sbjct  121 RGGDDDSEMSDGVLESGPVRMDVERHVVSVNGDTITLPLKEFDLLEYLMRNSGRVLTRGQ  180    Query  167 Y--------DISP-RTIEVYRANVMTKMQAN--SLSELVRLAMRAGM  202                     +   +T++V+   + +K++A+  +   LV  G  Sbjct  181 LIDRVWGADYVGDTKTLDVHVKRLRSKIEADPANPVHLVT-VRGLGY  226 |
| 5F64 | 4e-38 | Query  2 TTKGHIYVIDDDAAMRDSLNFLLDSAGFGVT-LFDDAQAFLDALPGLSFGCVVSDVRMPG  60          +   +  +IDD  ++LL  + +  + +  +  L V+ DV +PG  Sbjct  1 SNAMNAIIIDDHPLAIAAIRNLLIKNDIEILAELTEGGSAVQRVETLKPDIVIIDVDIPG  60    Query  61  LDGIELLKRMKAQQSPFPILIMTGHGDVPLAVEAMKLGAVDFLEKPFEDDRLTAMIESAI  120           ++GI++L+ ++ +Q I+I++   D      GA  F+ K + + A IE+A  Sbjct  61  VNGIQVLETLRKRQYSGIIIIVSAKNDHFYGKHCADAGANGFVSKKEGMNNIIAAIEAAK  120    Query  121 RQ--AEPAAKSEAVAQDIA--ARVASLSPRERQVMEGLIAGLSNKLIAREYDISPRTIEV  176                 P + +  V +   ++SLS +E  VM  ++ G  N  IA +  IS +T+  Sbjct  121 NGYCYFPFSLNRFVGSLTSDQQKLDSLSKQEISVMRYILDGKDNNDIAEKMFISNKTVST  180    Query  177 YRANVMTKMQANSLSELVRLAMRAGM  202           Y++ +M K++  SL +L   A R  +  Sbjct  181 YKSRLMEKLECKSLMDLYTFAQRNKI  206 |
| 4B09 | 6e-38 | Query  3 TKGHIYVIDDDAAMRDSLNFLLDSAGFGVTLFDDAQAFLDALPGLSFGCVVSDVRMPGLD  62               I +++D+  +   L   L +A +  TL   L  + ++ D+ +PG D  Sbjct  9 NTPRILIVEDEPKLGQLLIDYLRAASYAPTLISHGDQVLPYVRQTPPDLILLDLMLPGTD  68    Query  63 GIELLKRMKAQQSPFPILIMTGHGDVPLAVEAMKLGAVDFLEKPFEDDRLTAMIESAIRQ  122           G+ L + ++ + S  PI+++T   + +  +++GA D++ KP+ + A +++ +R+  Sbjct  69 GLMLXREIR-RFSDIPIVMVTAKIEEIDRLLGLEIGADDYIXKPYSPREVVARVKTILRR  127    Query  123 AEPAAK-------------SEAVAQDIAARVASLSPRERQVMEGLIAGLSNKLIAREYD-  168            +P  +                   ++  L+P E ++++ L       +  Sbjct  128 CKPQRELQQQDAESPLIIDEGRFQASWRGKMLDLTPAEFRLLKTLSHEPGKVFSREQLLN  187    Query  169 --------ISPRTIEVYRANVMTKMQANSLSELVRLAM  198                   ++ RTI+ +  N+  K+++ +A+  Sbjct  188 HLYDDYRVVTDRTIDSHIKNLRRKLESLDAEQSFIRAV  225 |
| 4YN8 | 5e-37 | Query  5 GHIYVIDDDAAMRDSLNFLLDS-AGFGVT-LFDDAQAFLDALPGLSFGCVVSDVRMPGLD  62             + +IDD   +R  L  +LDS V D   +   VV+D++MPG D  Sbjct  6 IRVMLIDDHPVVRAGLRSILDSFDDITVVAEASDGSN----INTKGIDVVVTDIQMPGTD  61    Query  63  GIELLKRMKAQQSPFPILIMTGHGDVPLAVEAMKLGAVDFLEKPFEDDRLTAMIESAI--  120           GI L + + A P+LI+T +  + A++ GA+ +L K   +  L   + +  Sbjct  62  GITLTRAL-ANAGGPPVLILTTYDTEADILAAVEAGAMGYLLKDAPESALHDAVVATFEG  120    Query  121 RQAEPAAKSEAVAQDIAARVASLSPRERQVMEGLIAGLSNKLIAREYDISPRTIEVYRAN  180           R+  + A+ Q ++ +LS RE ++++ L  GLSN+ +A + IS  T++ +  +  Sbjct  121 RRTLAPEVANALMQRVSKPRQALSAREIEILQNLEQGLSNRQLAAKLFISEATVKTHLVH  180    Query  181 VMTKMQANSLSELVRLAMRAGML  203           + +K+  ++ +  +  A +  ++  Sbjct  181 IYSKLGVDNRTAAITAARQQRLI  203 |
| 1P2F | 5e-36 | Query  6 HIYVIDDDAAMRDSLNFLLDSAGFGVTLFDDAQAFLDALPGLSFGCVVSDVRMPGLDGIE  65            I V+DDD  +   ++  L   G  V  F   + FL+ +F  VV DV +P   G E  Sbjct  4 KIAVVDDDKNILKKVSEKLQQLG-RVKTFLTGEDFLN--DEEAFHVVVLDVXLPDYSGYE  60    Query  66  LLKRMKAQQSPFPILIMTGHGDVPLAVEAMKLGAVDFLEKPFEDDRLTAMIESAIRQAEP  125           + + +K  + ++++T   D ++  + GA D++ KPF  + L A ++  + + +  Sbjct  61  ICRXIKETRPETWVILLTLLSDDESVLKGFEAGADDYVTKPFNPEILLARVKRFLEREKK  120    Query  126 A--------AKSEAVAQDIAARVASLSPRERQVMEGLIAGLSNKLIAREY-------DIS  170                    +  +  +  L  +E +++  L   +   +     +S  Sbjct  121 GLYDFGDLKIDATGFTVFLKGKRIHLPKKEFEILLFLAENAGKVVTREKLLETFWEDPVS  180    Query  171 PRTIEVYRANVMTKMQANS  189           PR ++ +   ++ +  Sbjct  181 PRVVDTVIKRIRKAIEDDP  199 |
| 1YS6 | 2e-35 | Query  18  DSLNFLLDSAGFGVTLFDDAQAFLDALPGLSFGCVVSDVRMPGLDGIELLKRMKAQQSPF  77            SL   L  +GF V D L +    +V D+ MP LDG+ ++  ++A  +  Sbjct  21  ASLERGLRLSGFEVATAVDGAEALRSATENRPDAIVLDINMPVLDGVSVVTALRAMDNDV  80    Query  78  PILIMTGHGDVPLAVEAMKLGAVDFLEKPFEDDRLTAMIESAIRQAEPAAKSEAVAQDIA  137           P+ +++ V   V  ++ GA D+L KPF L A +++ +R+ A S + +  Sbjct  81  PVCVLSARSSVDDRVAGLEAGADDYLVKPFVLAELVARVKALLRRRGSTATSSSETITVG  140    Query  138 ARVASLSPRERQVMEGLIAGLSNKLIAREYDISPRTIEVYRANVMTKMQANSLSELVRLA  197             +  R  +V  G+   L  RE+D+ E ++  V+++ Q   L  A  Sbjct  141 PLEVDIPGRRARV-----NGVDVDLTKREFDLLAVLAE-HKTAVLSRAQLLELVWGYDFA  194    Query  198 MRAGMLN  204             +++  Sbjct  195 ADTNVVD  201 |

*Table 2. PSI-BLAST alignments of FixJ_PDB_ (PDB ID 5xso, tetrahelical helix-turn-helix [HTH_4_] fold) with sequences of experimentally determined isolated winged helix (wH) folds (upper table). PSI-BLAST alignments of KdpEJ_PDB_ (PDB ID 4kfc, wH fold) with sequences of experimentally determined isolated HTH_4_ folds (lower table). In both tables, regions with differing secondary structures are bold.*

| **Query**  **Subject** | **Aligned query seq**  **Aligned subject seq** | **E-value** |
| --- | --- | --- |
| 5xso_A  1gxq_A | MIESAIRQAEPAAKSE**AVAQDI**AARVASLSPRERQVMEGLIAG----  -----LSNKLIAREYDISPRTIEVYRANVMTKMQANSLSEL**VRLAMRAGM**  AVEEVIEMQGLSLDPT**SHRVMA**GEEPLEMGPTEFKLLHFFMTHPERV  YSREQLLNHVWGTNVYVEDRTVDVHIRRLRKALEPGGHDRM**VQTVRGTGY** | 6.65e-04 |
| 5xso_A  1qqi_A | MIESAIRQAEPAAKSE**AVAQDI**AARVASLSPRERQVMEGLIAG----  -----LSNKLIAREYDISPRTIEVYRANVMTKMQANSLSEL**VRLAMRAGM**  AVEEVIEMQGLSLDPT**SHRVMA**GEEPLEMGPTEFKLLHFFMTHPERV  YSREQLLNHVWGTNVYVEDRTVDVHIRRLRKALEPGGHDRM**VQTVRGTGY** | 7.81e-04 |
| 5xso_A  4qwq_A | LSPRERQVMEGLIAGLSNKLIAREYDISPRTIEVYRANVMTKMQANS-**LSELVRLAMRAGM**  LASRENEVISK--SELLEKVWGYDYYEDANTVNVHIHRIREKLEKESF**TTYTITTVWGLGY** | 0.001 |
| 5xso_A  4u88_B | LSPRERQVMEGLIAGLSNKLIAREYDISPRTIEVYRANVMTKMQANS-**LSELVRLAMRAGM**  LASRENEVISK--SELLEKVWGYDYYEDANTVNVHIHRIREKLEKESF**TTYTITTVWGLGY** | 0.002 |
| 5xso_A  6kyx_A | **ESAIRQAEPAAKSEAVAQDIAARVASL**SPRERQVMEGLIAGLS-------  --NKLIAREYDISPRTIEVYRANVMTKMQANSLSEL**VRLAMRAGML**  **KDIIDVNGITIDKNAFKVTVNGAEIEL**TKTEYDLLYLLAENKNHVMQREQ  ILNHVWGYNSEVETNVVDVYIRYLRNKLKPYDRDKM**IETVRGVGYV** | 0.005 |

| **Query**  **Subject** | **Aligned query seq**  **Aligned subject seq** | **E-value** |
| --- | --- | --- |
| 4kfc_A  2rnj_A | **VDLAARVIHRGEEEVHL**TPIEFRLLAVLLNNAGKVLTQRQLLNQVWGPNAVEHSHYLRIYMGHLRQKLEQDPARPRHF  **VPRGSHMKKRAELYEML**TEREMEILLLIA----KGYSNQEIASA-----SHITIKTVKTHVSNILSKLEVQDRTQAVI | 2.64e-04 |
| 4kfc_A  7ve4_A | **VIHRGEEEVHL**TPIEFRLLAVLLNNAGKVLTQRQLLNQVWGPNAVEHSHYLRIYMGHLRQKLEQDPARPRHF  **MKKRAELYEML**TEREMEILLLIA----KGYSNQEIASA-----SHITIKTVKTHVSNILSKLEVQDRTQAVI | 0.004 |
| 4kfc_A  4wsz_B | **HL**TPIEFRLLAVLLNNAGKVLTQRQLLNQVWGPNAVEHSHYLRIYMGHLRQKLEQDPARPRHF  **DL**TNREHEILMLIAQGK----SNQEIADELF-----ITLKTVKTHVSNILAKLDVDDRTQAAI | 0.005 |

*Table 3 The transitive homology path of 5 sequences between FixJ_PDB_ and KdpE_PDB_. A diagram of the path is shown below.*

| Sequence A | Sequence B | Pairwise identity |
| --- | --- | --- |
| FixJ | WP_007679868 | 45% |
| WP_007679868 | PWU22963 | 38% |
| PWU22963 | TME68356 | 55% |
| TME68356 | WP_021443528 | 38% |
| WP_021443528 | HEK56308 | 43% |
| HEK56308 | KdpE | 48% |

48% ID

43% ID

38% ID

55% ID

38% ID

45% ID

**KdpE**

HEK56308

WP_021443528

TME68356

PWU22963

WP_007679868

**FixJ**

*Table 4. The 12 sequences in the branch containing the bridge sequences; the sequence also found in the transitive homology path (Supplementary Table 3; also called the “bridge sequence”) is highlighted in yellow.*

| GenBank Accession Number | Sequence |
| --- | --- |
| TMD58691.1 | MGESGPRAGVLLVVEDHVGVRSLVSLVLTAAGYVVREAASGEEAIEFARKEQPLLVLLDVRLPGISGYEVCGWLRDRFHDSVPVIFLSGERTEAFDRAAGLMLGADDYLVKPFSNEELVARVRGLLRRTLPAPRARGVGLTARELEVLRLLAGGLIQNDIAGHLLISTKTVGTHIEHILMKLGVQSRAQAVALAYRDNLIEPNAPLPIAPSASGPATLSNGAHGDASVSFRTTTAPKPAR |
| TMC70452.1 | SLITAGEPKRSADPNLRAHVQSPLSRAPCAASGVIAGRWDVSGRDGQRRHAVLVVEDDIEVRSLITDVLTHAGYIVRGVQSGEEAIDSVRQEDPILVLVDVHLPGMSGYEVCGWLRSRYRDSVPVMFISGERVEAFDRAAGLMLGADDYMSKPFSTEELVARVRGLLRRTVPPGQSLDTKLTVRELEVLRLLAGGLGQKEISGHLSISAKTVGTHIEHILMKLGVQSRTQAVALAYRERLIDAEDEGESEVRGPTSAKPTS |
| TME68356.1 | MARTVLAWAPTRRSLKRPGTRHGGTRVASSSPRGTILLVDDDPDIRLLLKTVLCNAGFATNEAVSGEAAVETMRREQPLLVVLDVRLPGVSGYEVCRWLRERFRDIVPIIFMSAERKESFDRAAGLMLGADDYMMKPFSVDELIARIRGLLRRTVPSPRLLAESLTARELEVLRLLAGGLTQADIANQLLISGKTVGTHIEHILMKLDVPSRAQAVAVAYRENLVEPTVAMPTITLTTASRPSSFGASLLPAATLRMASVAKAAR |
| TMC70453.1 | MSAGGMTVGGPGPRGAILVVEDDADIRSLLSTVLTGAGFAVNTVASGEAAVELMRREQPVLVLLDIRLPGLSGYEVCRWLRERFRDVVPIVFMSAERKESFDRAAGLMLGADDYLMKPFLNEELLARIRGLLRRTVPTPRLLAENLTARELEVLRLLAGGLTQADIANQLLISGKTVGTHIEHILMKLDVPSRAQAVAVAYRENLVEPNGAAPVGPWSTRRVPPTFGSTAVPPATLRLSGQTKSARSG |
| TMB79520.1 | MSELVLVVDDDPNVRGLIVNLLSDYGFETAEASCGEEALRFARERPPDVVLLDVLMPGLSGYEVLRKLKDDFGDSVGVILISGERRESYDRVAGLLVGADDYLPKPFALDELLARVQKLVRGKRRGLPGKRRLTRRELEVLAHLVAGLDNAQIAQRLVLSPRTVTTHVEHILMKLGVHSRTQAIAMAYREGFFEGPSLRVIEGELAAAE |
| OLE96773.1 | MLVVDDDPVICDLVATTLADQGYATRRASDAREALHLIELETPDVVLLDVHLPDLSGYQLCRRLRDTLGDTMGIMLISGERREAFDRAAGLLLGADDYLVKPFVLDELLARVHRMAQRARPVTLSVAARLTRREAQVLRMLAAGLEQKDIARDLVVAPRTIAKHIEHILLKLGVHSQAQAIALAFRTELAGAVTPHDREEIRVEGT |
| TMC42982.1 | MREGGASMGNPILIVDDDSIIRAAIATILADAGYATREADSGAEALRVARSDAPGLVLLDVNLPGMCGYEVCRLLRDEFGDQFPIVFVSGARTESFDRVAGLLLGANDYISKPFREDELLARVQSLLLRRHAVASRALASRLTARELQVLRLLSTGLGPDDIARLMVISPKTVGAHVEHIYMKLGVQTRAQAVAVAYRGELLNGETAPTLEPSGNTSR |
| TML48806.1 | MAAATHDGRFVLVVDDDDTFRSLVSEILHRGGYRTRGAATGEEALRVARKKRPSFVLLDVNLPGMSGYDVCRELRAEFGEQLPIVFVSGERTERFDRVAGLRLGADDYIVKPFDPSELLARVDRFVGRAQAIFSEAATSPFRLTKRELEVLDYLVRGFTPKETARELTISRKTVATHIQNILTKLDVHSQAQAVAVALQSSLFDFSTRDSESGERSRAHSR |
| TML69799.1 | MTIDCGRGGILVIDSDDEGRAVVSALLADAGYITREAATGREGMSAARRERPDLVLLEVELRDMTGYEVCRQLRDAYGEGLPVIFVAATRTGSADRIAGLLIGADDYVVKPFAPDELIARVRRALIRTMSLSRHRNGSRSSYGLTEREREVLRLLSQGLPQKTIARELFISPQTVATHIQRTLAKLDVHSRAEAVALAHRERLVDDGVGLSAVPEAAP |
| TML69678.1 | MRYNLGAHRRGAQSGERGRVVDCGTVLVVDADDVWRSATAALLTRVGHDTIGAATAADALELARRERPSLVVLDVALPDLDGYEVCRELRDVYGDKLPIILVSADRNEPHDCIAALLIGADDYLAKPYNPGELLARVHRHLTRTNGSNDLTQPKPNTDGSDGFGLSPRELEVLRLLASGLDQAAIAQELVISPKTVSSHIQRILAKLGVHSRAQAVAVAHQEGLVADFAAHAVVFAAPAPSG |
| PWU22963.1 | MRGPILIVDDDPATRELVACTLGSAGYDTLELPTGEQALIAAGEERPALVVLDVKLPGVSGYEVCRQLRERFGEQLPILFVSGERAEAHDRVAGLMIGADDYVTKPFLPDELLARVGRLLVRAKPPAVAADDDLERWAQLTDREREVLNLLAEGLSQDAIAERLYISPKTVATHIQRILAKLGVHSRAAAVSRAYRLGLVSPDFATHMFAFSDA |

*Table 5. Ancestral sequences reconstructed from the consensus phylogenetic tree (Supplementary Figure 5)*

| *Name* | *Sequence* |
| --- | --- |
| Anc0 | TATASMLARVLVVDDDPAIRELLADALEREGYRLVVAATGEEALEAAVRGEQRPDLVLLDLALPDLDGFEVCRQLRQQRSNSVPVLFLTGRGEEDDKLRGFRAGADDYVTKPFGPEELLARIRALLRRSRATSPAAAAPSLADAGDDLTLDPARQQATLGLRQPAGLTPREIDLLRLLAQGPGRVVSNREIGEQLGGSELEDADAARKTVETHVQRLLQKLGADAVSDSRAGAAVQAVAYRLAGSSPSG |
| Anc1 | TSATSPRARILIVDDDPAIRDMLRDALDREGYEVVVAASGEAALEAAVRQEQRPDLVLLDVMLPGMDGFEVCRQLRDQRSNSVPIIFLTGHGDEDDRVAGLRVGADDYVTKPFSPDELLARIRALLRRSRPTRPAAAAPSALRAGDDLSLDPAQQEATLGLQQPAGLTPRETEVLRLLAQGPGRVVSNKEIAEQLGVGEYEDADAATRTVQVHVRRILKKLGDDGVSHSRAAAVVRGVAYRLAAPSPPS |
| Anc2 | GATASMRARILVVDDDPAIREALRDALERAGYELVTAASGEEALEMAVRNEQQPDLVLLDVMMPGLDGFEVCRQLRQQRSNSVPIIFLTGHGEEDDRVAGLQAGADDYVTKPFSPDELLARIRALLRRSRAASPAAAAPSVLDAGDDLTLDPARQQATLGLQQPAGLTPRETELLRFLAQGPGRVVSNKEIAEQLGISEYEDADAATRTVQTHVQRLLQKLGDDGVSHSRAAAAVRALAYRLGASSPSS |
| Anc3 | TSATSMMARILIVEDDPAIREMLRDALEREGYEVTTAASGEAALEAAVRQEQRPDLVILDVMLPGMDGFEVCRRLREQSSNSVPIIMLTGRGEEADRVAGLELGADDYVTKPFSPAELLARIRAVLRRSSPAAPAAAPSSVLRFGDDLTLDPARREVTLGAGQPVELTPKEFELLAFLAQRPGRVFSREEILEQVWGPEYEDADAATRTVEVHISRLRKKLGDNSPSNPRYLLTVRGVGYRFAAPSSES |
| Anc4 | GAAASGRGAILVVDDDPAVRSLIAAILARAGYRLREAATGEEALEAAARGEQRPDLVLLDVRLPGLSGYEVCRQLRDQRGDQLPIIFVSGERESDDRVAGLLIGADDYLTKPFSPDELLARVRRLLRRTHAASPAAAAPSLADAGDDLTLDAPRNDDTLGLSRAAGLTPREREVLRLLAQGPARVLSQKEIAQQLVISPLEDADAATKTVRTHVQRILAKLGDDGVAHSRAQAVVRALAYREGLVDPDS |
| Anc5 | GGSGPRRGAILVVEDDPDIRSLLSTVLTRAGFRLNEAASGEAAVELMARGEQRPLLVLLDVRLPGLSGYEVCRWLREQRRDVVPIIFMSAERESDDRAAGLMLGADDYLMKPFSNEELLARIRGLLRRRTVPSPAAAAPSLADAGDDLALDSPRNDGTLGLSLAENLTARELEVLRLLAQGPARVLSQADIANQLLISGLEDADAARKTVHTHVQRILMKLDDDAVAPSRAQAVVRAVAYRENLVEPNA |

**References**

1 Eddy, S. R. A new generation of homology search tools based on probabilistic inference. *Genome Inform* **23**, 205-211 (2009).

2 Edgar, R. C. MUSCLE: multiple sequence alignment with high accuracy and high throughput. *Nucleic Acids Res* **32**, 1792-1797, doi:10.1093/nar/gkh340 (2004).

3 Ashkenazy, H. *et al.* ConSurf 2016: an improved methodology to estimate and visualize evolutionary conservation in macromolecules. *Nucleic Acids Res* **44**, W344-350, doi:10.1093/nar/gkw408 (2016).

4 Sievers, F. *et al.* Fast, scalable generation of high-quality protein multiple sequence alignments using Clustal Omega. *Mol Syst Biol* **7**, 539, doi:10.1038/msb.2011.75 (2011).

5 Altschul, S. F. *et al.* Gapped BLAST and PSI-BLAST: a new generation of protein database search programs. *Nucleic Acids Res* **25**, 3389-3402, doi:10.1093/nar/25.17.3389 (1997).
