# Supplementary material for: Identification of a covert evolutionary pathway between two protein folds
Source: Nat Commun. 2023 Jun 1;14:3177. doi: 10.1038/s41467-023-38519-0 (PMC10235069; doi:10.1038/s41467-023-38519-0)
Supplement: Supplementary file 4 — Description of Additional Supplementary Files [file 41467_2023_38519_MOESM4_ESM.pdf]

Description of Additional Supplementary Files

Supplementary Data 1
Description: Cross-family sequence alignments of helix-turn-helix and winged helix proteins.

Supplementary Data 2

Description: PSI-BLAST and jackhmmer searches of C-terminal domain sequences against the Protein Data Bank.

Supplementary Data 3
Description: Lists of Protein Data Bank entries that are homologous to response regulators with helix-turn-helix and winged helix C-terminal domains.
